# Emerging Applications of Thiol-Based Catalysts in Hydrogen Atom Transfer Reactions: A Comprehensive Review

**DOI:** 10.3390/molecules30204058

**Published:** 2025-10-11

**Authors:** Hao Yang, Yanyan Liao, Hao Guo, Ming Wang

**Affiliations:** 1 School of Chemistry and Molecular Engineering, East China Normal University, 3663 North Zhongshan Road, Shanghai 200062, China; haoyang_jianggroup@163.com (H.Y.); yyliao@chem.ecnu.edu.cn (Y.L.); 2 Ecological Environment Monitoring and Scientific Research Center, Yellow River Basin Ecology and Environment Administration, Ministry of Ecology and Environment, Zhengzhou 450004, China

**Keywords:** hydrogen atom transfer, thiols/thiophenols, photocatalytic

## Abstract

Hydrogen atom transfer (HAT) is a fundamental class of radical transformations that enables the direct generation of open-shell radical intermediates from R–H bonds (R = C, N, etc.), offering unique opportunities for green and sustainable synthesis. Significant progress has been made not only in identifying diverse molecular scaffolds capable of mediating HAT but also in developing synthetic methodologies to achieve precise stereocontrol in these processes. In this context, this review highlights recent advances in the use of sugar-derived compounds, cysteine-containing peptides, and chiral/achiral thiols/thiophenols as catalysts for stereoselective HAT, emphasizing their potential to expand the synthetic utility of HAT in organic transformations.

## 1. Introduction

**Scheme 1:**
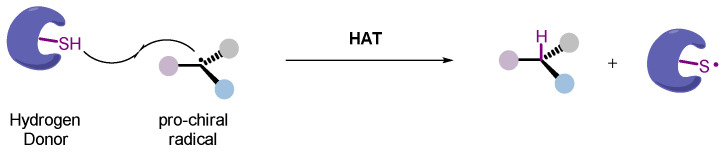
Thiol-involved asymmetric hydrogen atom transfer (HAT).

The HAT process plays a fundamental role across various chemical domains, including hydrocarbon transformations, atmospheric chemistry, and enzymatic catalysis [1,2,3,4]. Mechanistically, HAT involves the concerted movement of one proton and one electron (H = e^−^ + H^+^) from a hydrogen donor substrate to a hydrogen abstractor in a single kinetic step (Scheme 1) [5,6,7,8]. This strategy has emerged as a powerful approach for C-H bond activation. Unlike conventional metal-catalyzed C-H activation methods, the HAT mechanism offers distinct advantages: it eliminates the requirement for pre-installed directing groups, proceeds under mild and operationally simple conditions, and often exhibits selectivity profiles that differ from those observed in traditional metal-mediated processes [9,10,11,12].

Over the past few decades, significant research efforts have focused on developing an intermolecular HAT process through various approaches, including metal–organic frameworks (MOFs) [13], transition metal-based systems [14,15,16], photocatalysis [17,18,19,20,21,22], and enzymatic catalysis [23,24,25]. While considerable progress has been made in developing hydrogen atom donors featuring heteroatom- or metal-bound hydrogen (such as stannanes, silanes, transition metal hydride complexes, and thiols), alkanethiols exhibit nearly identical behavior in hydrogen-atom abstraction owing to their comparable S–H bond dissociation energies (approximately 87 kcal mol^−1^) [26]. In contrast, thiophenols act as excellent hydrogen-atom donors, since the resulting arenethiyl radicals are stabilized through resonance. When coupled with a judiciously designed chiral environment proximal to the sulfur center, these systems enable effective stereocontrol over the reaction products [27]. Early work by Roberts’s group demonstrated the potential of chiral control in these reactions, employing sugar-derived chiral thiols to achieve radical chain hydrosilylation of alkenes with silanes under thermal conditions [28]. Notably, this strategy yielded hydrosilylation products with up to 95% enantiomeric excess (ee) when the substrate is 6-methylene-5,5-diphenyltetrahydro-2*H*-pyran-2-one. Recent advances have revealed that cysteine-containing peptide compounds can serve as effective hydrogen atom donors (HAT donors). These systems offer particular advantages, as their well-defined secondary structures facilitate chiral transfer, while the modular nature of peptides enables precise tuning of reactivity and selectivity through strategic amino acid substitution. Photocatalytic approaches have emerged as particularly promising, leveraging light’s mild and clean characteristics [29]. Several research groups have successfully achieved asymmetric HAT reactions using chiral thiophenols as HAT donors under photocatalytic conditions, establishing an attractive platform for stereocontrolled transformations [30,31].

**Scheme 2:**
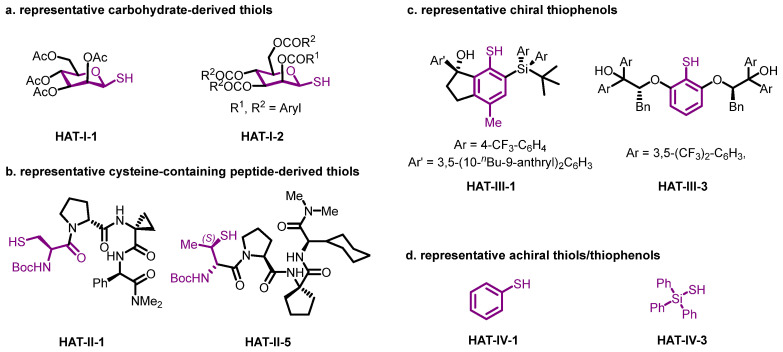
Representative thiols and thiophenols.

Although thiol/thiophenol-mediated HAT reactions have been explored, controlling the stereochemistry of HAT reactions remains a formidable challenge. This difficulty primarily stems from the planar geometry of radical intermediates and their rapid configurational inversion. This review provides a comprehensive summary of HAT processes mediated by chiral thiols/thiophenols and their derivatives (Scheme 2), including relevant mechanistic studies. Representative examples of HAT reactions involving achiral thiophenols as hydrogen donors are also presented, offering readers a clearer perspective on the intrinsic challenges in stereocontrol. Metal-based systems [14], and enzymatically catalyzed HAT [23] are explicitly excluded. Our objective is to highlight current advancements and outline future challenges in this field, thereby establishing a theoretical foundation for the development of novel thiol-mediated HAT methodologies.

## 2. Hydrogen Atom Transfer Reagent of Chiral Thiols/Thiophenols

### 2.1. Hydrogen Atom Transfer Reagent of Sugar-Derived Thiols

A groundbreaking advancement in asymmetric organocatalysis involves the use of carbohydrate-derived thiols as protonative polarity-reversal catalysts to enable enantioselective C–H bond formation. The high efficacy of polarity-reversal catalysts is attributed to two main factors: first, the thiyl radical exhibits relatively high electrophilicity, which facilitates hydrogen abstraction from hydrosilane; second, the presence of multiple electronegative oxygen atoms enhances the strength of the S–H bond. This strategy employs only a catalytic amount of thiol as the polarity reversal catalyst.

**Scheme 3:**
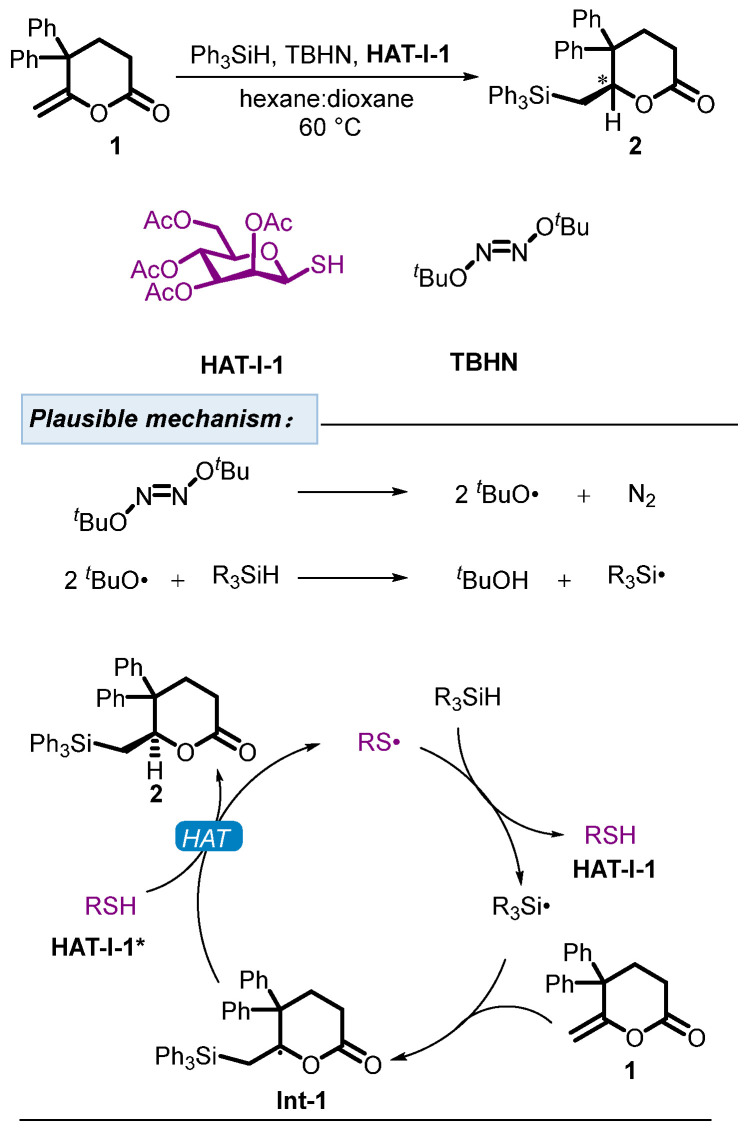
Asymmetric hydrosilylation using carbohydrate-derived thiols. * denotes a chiral center.

Roberts and co-workers pioneered the design, synthesis, and catalytic application of novel homochiral carbohydrate-derived thiols—bearing a sulfhydryl group at the anomeric carbon—as protic polarity-reversal catalysts. These thiols facilitate the enantioselective radical-chain hydrosilylation of prochiral methylenelactones with triphenylsilane (Scheme 3) [28]. Mechanistically, the thiol catalysts initiate a radical chain process: (1) initiation generates a Ph_3_Si• radical, which adds to the exocyclic methylene group of the lactone substrate, forming a prochiral α-silylalkyl radical intermediate (eg, **Int-I**); (2) this intermediate then undergoes enantioselective HAT from the chiral thiol catalyst. The key innovation lies in the thiol’s role as a polarity-reversal catalyst. Direct HAT from the silane to the nucleophilic alkyl radical (**Int-I**, formed via silyl radical addition to the alkene) is thermodynamically disfavored. However, the electron-deficient hydrogen of the thiol catalyst enables efficient HAT from the silane to the β-silyl alkyl radical **Int-I**, because the electron-deficient hydrogen atom is more readily abstracted by the electron-deficient β-silyl alkyl radical, thereby reducing the energy barrier for hydrogen atom transfer, overcoming this limitation and ensuring high enantioselectivity.

**Scheme 4:**
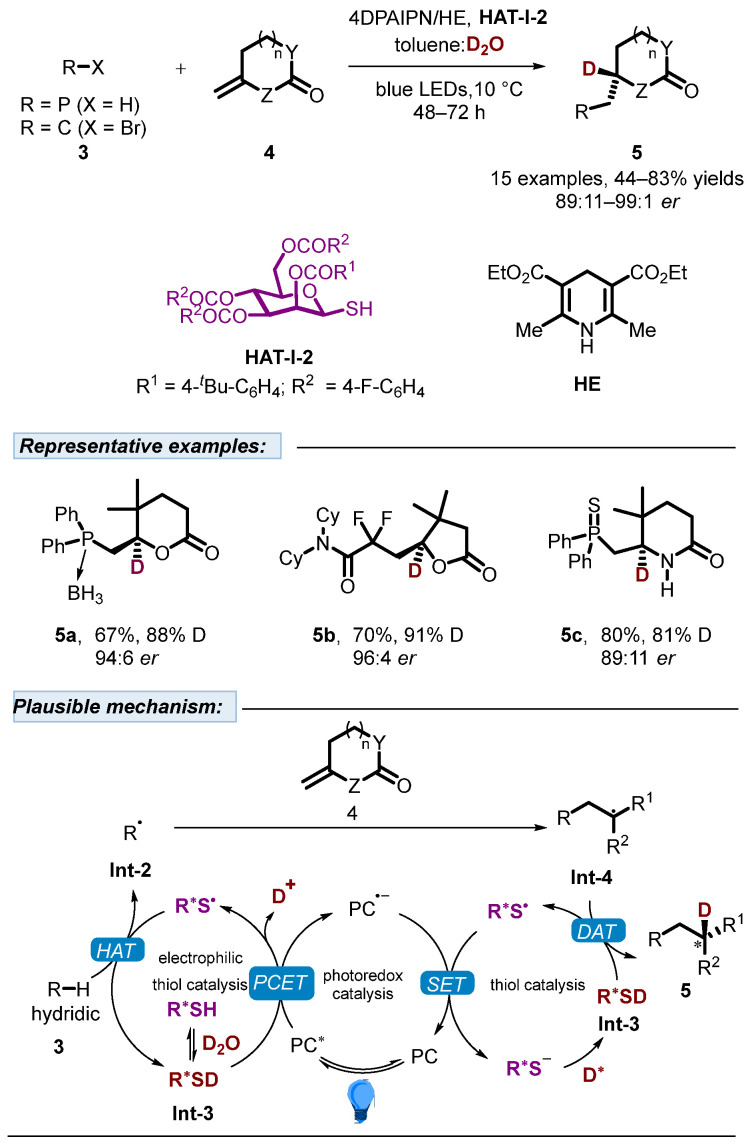
Asymmetric deuteration by carbohydrate-derived thiols. * denotes a chiral center.

In 2022, Ye’s group and Funes-Ardoiz’s group developed a novel visible-light-mediated catalytic asymmetric radical deuteration strategy (Scheme 4) [32]. The key innovation involves employing a rationally designed mannose-derived thiol catalyst **HAT-I-2** (possessing multiple hydrogen bond sites) in conjunction with cost-effective D_2_O as the deuterium source; this method achieved deuterophosphinoylation and deuterodifluoroalkylation of exocyclic olefins. Notably, it overcomes the limitation of traditional two-electron mechanisms restricted to benzylic deuteration, delivering products with excellent enantioselectivity (up to >99:1 er) and high deuterium incorporation (up to 97%). The key innovation involves employing a rationally designed mannose-derived thiol catalyst **HAT-I-2** in conjunction with cost-effective D_2_O as the deuterium source within a photoredox catalytic system.

The proposed mechanism involves the following steps: (1) Visible-light excitation of a photoredox catalyst (PC) generates its excited state (PC*), which oxidizes the thiol catalyst via a concerted proton-coupled electron transfer (PCET) process in the presence of D_2_O. This step produces an electrophilic thiyl radical species and the reduced (PC^•–^). (2) HAT between the thiyl radical and a hydridic R_1_–H bond (R1 = B, Si) of the substrate forms radical species **Int-2**. (3) Addition of **Int-2** to the olefin generates a nucleophilic carbon-centered radical species **Int-4**. (4) Driven by polarity matching (an electrophilic radical combines with a nucleophilic radical), the tertiary alkyl radical **Int-4** abstracts a deuterium atom from the electrophilic thiyl radical species. **Int-4** undergoes an asymmetric deuterium atom transfer (ADAT) with the in situ-generated deuterated chiral thiol (**Int-3**), completing the enantioselective deuteration.

**Scheme 5:**
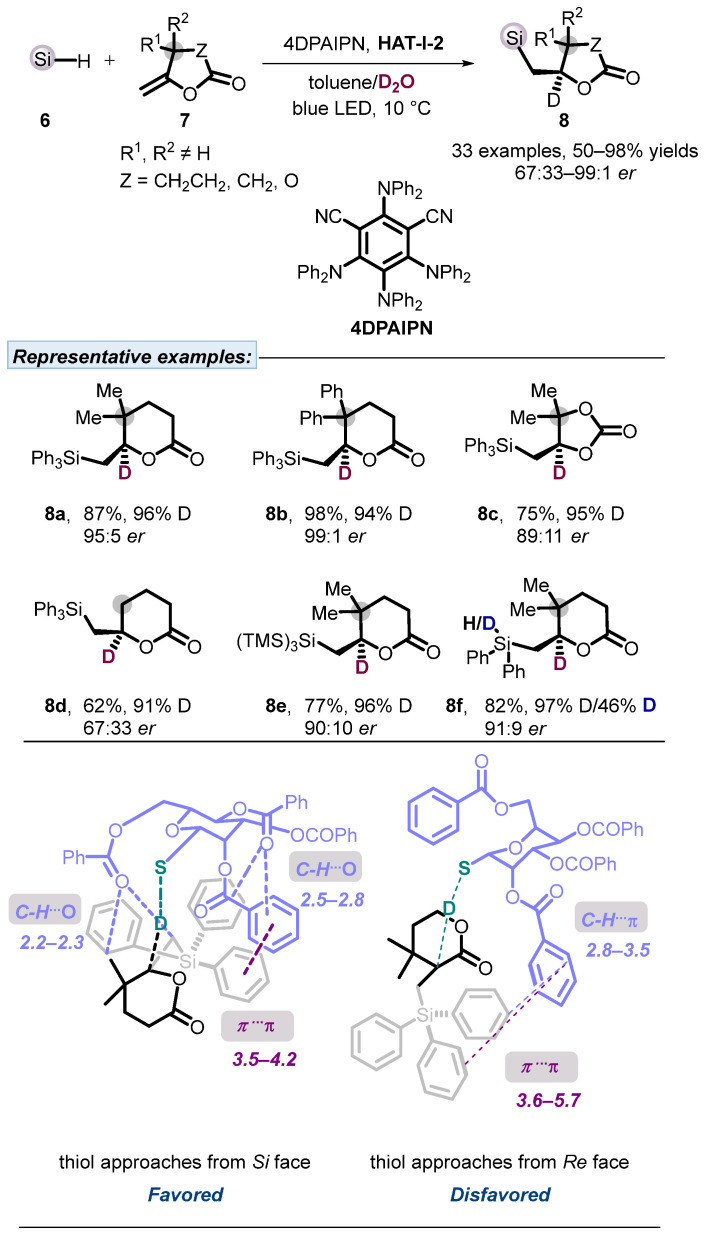
Asymmetric deuteration by carbohydrate-derived thiols.

In 2023, Ye and colleagues reported a visible-light-driven catalytic asymmetric deuterosilylation of exocyclic olefins bearing allylic substituents. This transformative methodology achieves exceptional enantioselectivity (up to >99:1 er) and remarkable deuterium incorporation (>90% D) across a broad substrate scope (Scheme 5) [33]. The proposed mechanism involves photoexcitation of an organic photocatalyst (eg, **4CzIPN**) initiating a concerted PCET process with the thiol catalyst to generate a thiyl radical. This radical subsequently abstracts a hydrogen atom from the silane reagent, forming a silyl radical that adds across the exocyclic olefin to produce a prochiral carbon-centered radical intermediate. Enantiocontrol is established through a stereodetermining deuterium atom transfer (DAT) from the deuterium-labeled thiol (-SD) to thiyl radical intermediate (-S•). Density functional theory (DFT) calculations reveal that the exceptional stereoselectivity stems from multiple favorable non-covalent interaction (NCI), including π-π stacking and C-H···O hydrogen bonding between the catalyst and prochiral radical adduct in the preferred transition state, rather than through steric differentiation. This mechanistic paradigm contrasts sharply with conventional thermal approaches and successfully addresses long-standing challenges in achieving high enantioselectivity for allylic-substituted systems.

### 2.2. Hydrogen Atom Transfer Reagent of Peptide-Derived Thiols

Peptide catalysts offer unique advantages in multifunctional substrate activation and address selectivity challenges across diverse stereochemical regimes, including point, axial, and conformation-dependent stereochemistry. In biological systems, cysteine-mediated processes are ubiquitous, where active-site thiyl radicals play pivotal roles in chemical transformations. For example, thiol residues play an important role in essential deoxygenation of ribonucleotides [34], a transformation shared by all living organisms in the de novo synthesis of DNA precursors [35].

**Scheme 6:**
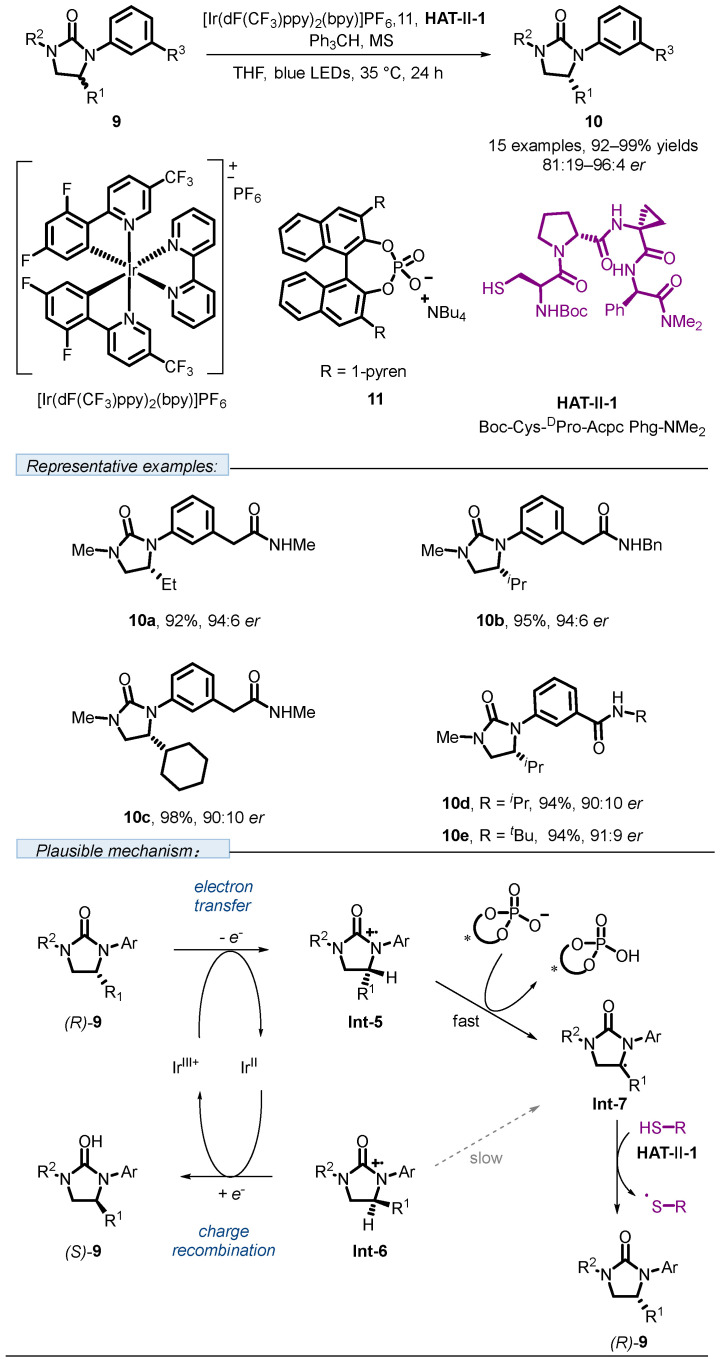
Light-driven deracemization of ureas. * denotes a chiral center.

In 2019, Knowles’s group and Miller’s group reported a light-driven deracemization strategy for cyclic ureas, overcoming intrinsic thermodynamic constraints (ΔG = +0.42 kcal/mol) via excited-state electron transfer mediated by a triple-catalyst system. This approach combines visible-light irradiation with an Ir (III) photoredox catalyst, a chiral BINOL-derived phosphate Brønsted base, and a cysteine-containing peptide thiol HAT catalyst (Scheme 6) [36]. Mechanistically, photoexcited *Ir(III) undergoes single-electron transfer (SET) to the racemic urea substrate, generating a prochiral α-amino radical cation. The chiral phosphate then facilitates enantioselective deprotonation at the acidified stereogenic methine C–H bond, yielding a configurationally labile α-amino radical intermediate **Int-7**. Subsequent diastereoselective HAT from the chiral thiol catalyst reforms the C–H bond with inverted stereochemistry. Finally, PCET between reduced Ir(II), the thiyl radical, and protonated phosphate regenerates all catalytic species. Key to the system’s success is the multiplicative enantioselectivity arising from two sequential stereodetermining steps: enantioselective deprotonation (er_PT_) and diastereoselective HAT (er_HAT_), giving an overall enantiomeric ratio er_obs_ = er_PT_ × er_HAT_. This synergy effect enables superior selectivity (up to 96:4 er) compared to either step individually. The reaction achieves near-quantitative yields and high enantioselectivity across 15 substituted cyclic ureas, establishing a catalyst-controlled non-equilibrium state without stoichiometric reagents. This work represents a distinct photoredox deracemization paradigm that diverges from energy-transfer mechanisms, instead leveraging cooperative asymmetric catalysis to circumvent microscopic reversibility limitations.

**Scheme 7:**
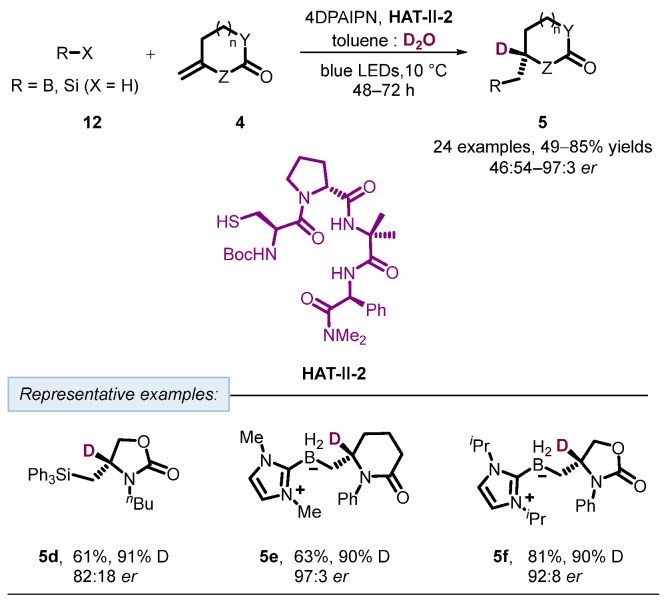
Asymmetric deuteration by peptide-derived thiols.

In 2022, Ye et al. and Funes-Ardoiz et al. reported a visible-light-mediated catalytic asymmetric radical deuteration strategy (Scheme 7) [32]. Expanding on previous work with peptide-derived thiol catalysts, this study successfully employed **HAT-II-2** in combination with D_2_O as the deuterium source. The system enabled both deuteroboration of exocyclic olefins and deuterosilylation of alkenes, achieving exceptional enantioselectivity (up to >99:1 er) and high deuterium incorporation (up to 96%). These results demonstrated the broad utility of this approach for asymmetric radical deuteration at non-benzylic positions.

**Scheme 8:**
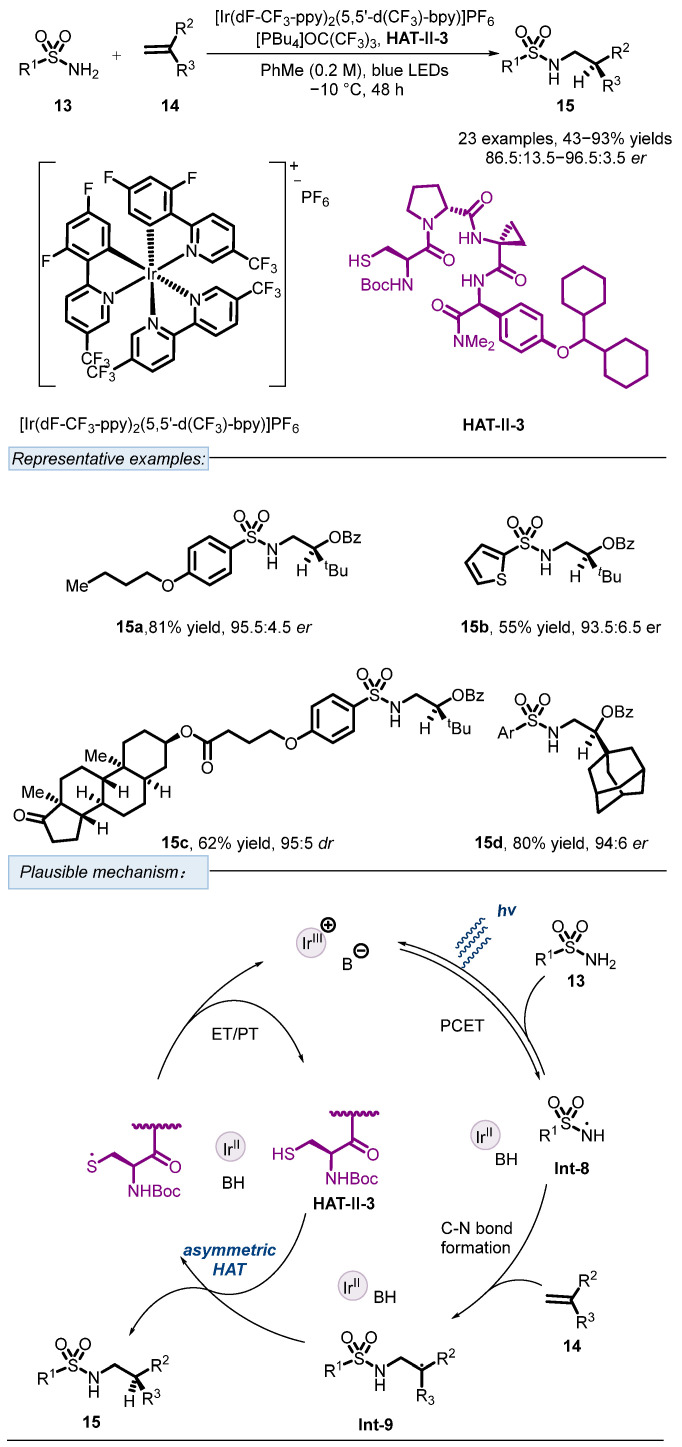
Tetrapeptide thiol-catalyzed hydroamination.

A significant advance came in 2025 when Knowles, Miller, and Houk et al. developed a highly enantioselective radical hydroamination of enol acetates/benzoates with sulfonamides. Their synergistic triple catalytic system combined an Ir(III) photoredox catalyst, a Brønsted base, and a novel chiral tetrapeptide thiol catalyst (eg, Boc-Cys-^D^Pro-Acpc-Phg-NMe_2_, **HAT-II-3**) (Scheme 8) [37]. The mechanism involves photoinduced PCET-mediated N–H homolysis of the sulfonamide, followed by anti-Markovnikov radical addition to form key intermediate **Int-9**. Enantiocontrol was achieved through stereoselective HAT from the peptide catalyst’s cysteine thiol moiety. Structural optimization revealed that a β-turn-biased tetrapeptide scaffold with a C-terminal phenylglycine residue (**8**) provided optimal performance, delivering 23 β-amino alcohol products with up to 97:3 er. Mechanistic studies, including Hammett analysis and kinetic measurements (showing 2.2-fold rate acceleration with **HAT-II-3**), combined with computational modeling, identified a cooperative network of noncovalent interactions (NCIs) governing stereocontrol: hydrogen bonding, π–π stacking, and crucially, London dispersion forces between catalyst and substrate. This work represents a landmark in asymmetric HAT catalysis with small molecules, providing new insights into stereocontrol at open-shell intermediates.

**Scheme 9:**
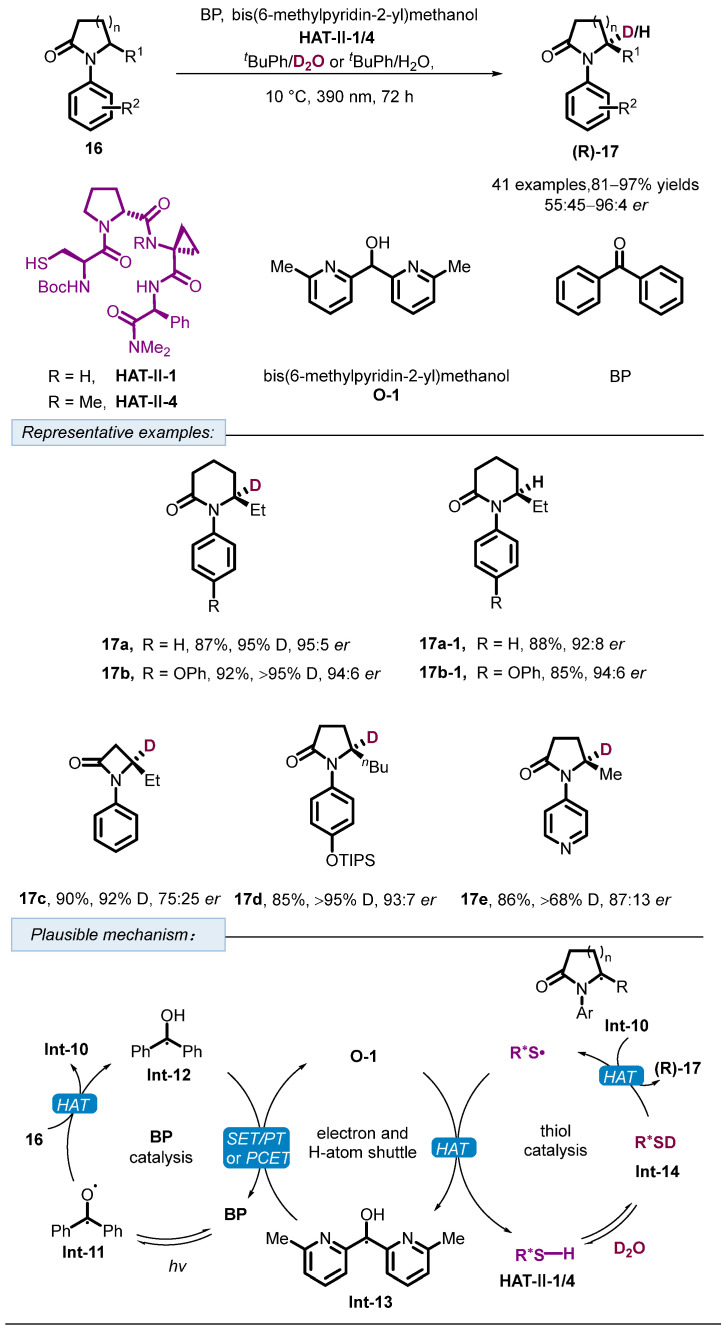
Photochemical deracemization of lactams with deuteration enabled by peptide-derived thiols. * denotes a chiral center.

Ye et al. recently reported an innovative photochemical deracemization strategy for δ- and γ-lactams featuring tertiary stereocenters (Scheme 9) [38]. Their dual HAT system employs benzophenone (BP) as a non-selective hydrogen atom abstractor to generate prochiral α-amino radical intermediates **Int-10** via homolytic C(sp^3^)–H cleavage at 390 nm, followed by enantioselective HAT from chiral thiol catalyst **HAT-II-1/4**. Key to the system’s success was bis(6-methylpyridin-2-yl)methanol as a multifunctional additive that both facilitates BP turnover (preventing benzopinacol formation) and enhances HAT enantioselectivity through hydrogen-bonding. The method achieves excellent deracemization and site-selective deuteration (>95% D incorporation from D_2_O) with enantiomeric ratios up to 96:4 er, and has been applied to synthesize enantioenriched deuterated natural products.

**Scheme 10:**
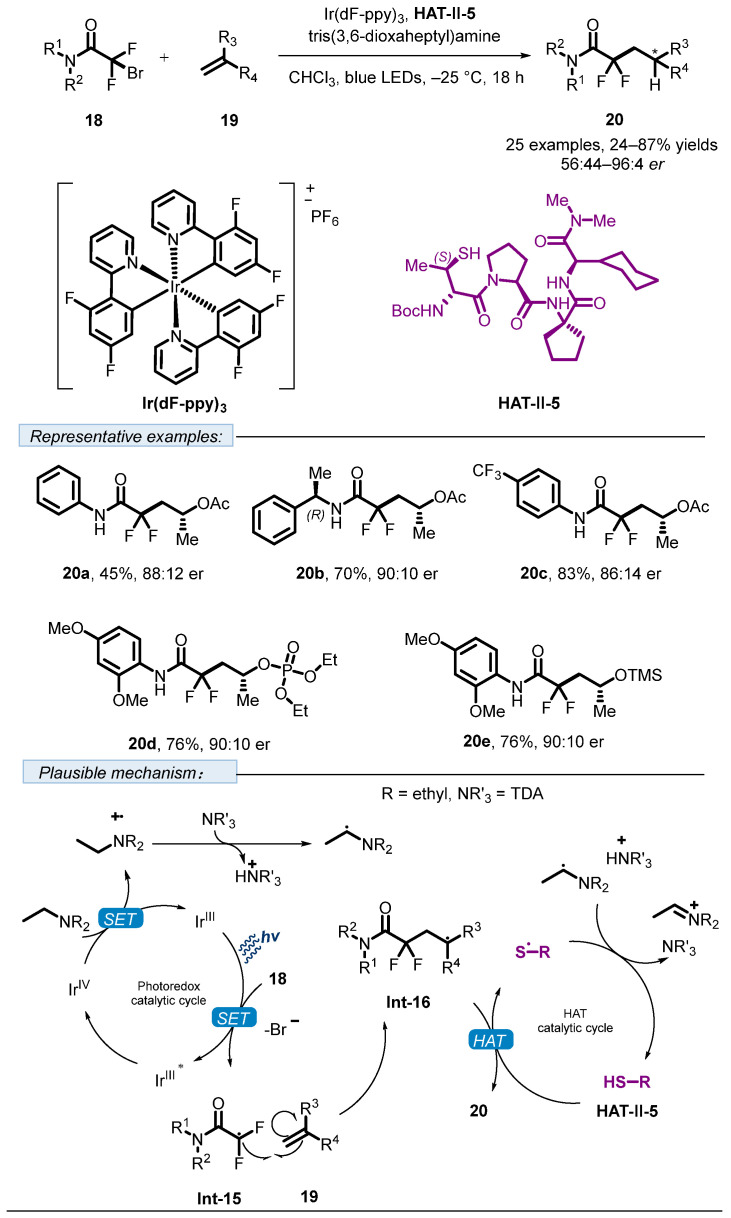
Peptide-derived thiols catalyzed Hydrodifluoroalkylation of alkenes. * denotes a chiral center.

Concurrently, Miller et al. reported an enantioselective hydrodifluoroalkylation of alkenes using conformationally constrained thiol-containing tetrapeptide catalysts (Scheme 10) [39]. The transformation involves photoredox generation of difluoroacetyl radical from bromodifluoroacetamide, followed by anti-Markovnikov addition and stereo-determining HAT. Key to the high enantiocontrol (up to 96:4 er) was the strategic incorporation of an (*S*)-*β*-methylcysteine residue at the N-terminus, which was crucial, enforcing a catalytically active type II’ β-hairpin conformation (verified by X-ray crystallography) while suppressing unproductive thiyl radical pathways. With Ir(dFppy)_3_ as photocatalyst and tris(3,6-dioxaheptyl)amine as terminal reductant, the system achieved up to 96:4 er across diverse substrates, including pharmaceutically relevant compounds. DFT studies revealed that enantioselectivity arises from stabilizing NCls and reduced distortion energy in the favored HAT transition state, with the β-methyl group playing a critical conformational control role.

### 2.3. Hydrogen Atom Transfer Reagent of Chiral Thiophenols

While mannose-derived chiral thiols, cysteine-containing peptides and tributyltin hydrides have emerged as effective HAT reagents, their capacity to relay stereochemical information from the hydrogen donor to prochiral radical center remains limited in certain transformations. Thus, the development of general and efficient chiral HAT catalysts featuring a densely structured chiral pocket around the reactive hydrogen to achieve high enantioselectivity continues to pose a significant challenge.

**Scheme 11:**
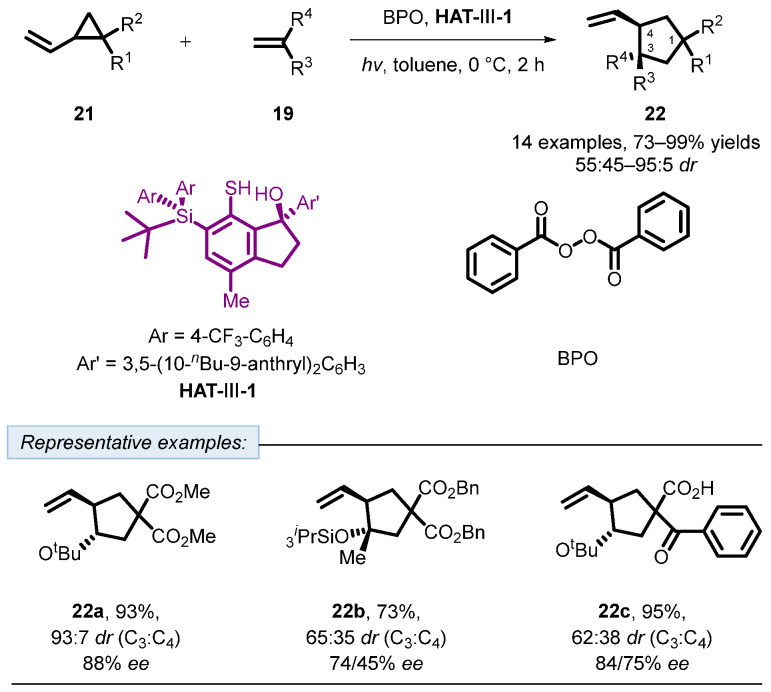
Chiral thiyl radical catalyzed addition and cyclization.

In 2014, Maruoka’s group introduced a chiral thiol precatalyst (**HAT-III-1**) for enantioselective radical cyclization (Scheme 11) [40]. The catalyst, built on an indanol scaffold, incorporates a sterically encumbered chiral pocket through a diaryl(tert-butyl)silyl group and a bulky 10-*tert*-butyl-9-anthryl substituent, shielding three quadrants around the catalytic sulfur center. Upon photolytic or oxidative generation, the corresponding thiyl radical adds to an electron-deficient vinylcyclopropane (**21**), triggering ring-opening to form an alkyl radical intermediate. Subsequent addition to a vinyl ether (**19**) and intramolecular cyclization—the stereodetermining step—occurs within the confined chiral environment, delivering products with high diastereo- and enantioselectivity (up to 95% dr, 87% ee). The catalyst is regenerated via HAT, completing the catalytic cycle. This work marked a paradigm shift from conventional acid-base organocatalysis, demonstrating the first highly enantioselective radical C–C bond formation under mild conditions, even tolerating polar functionalities (e.g., free carboxylic acids) at low catalyst loadings (1 mol%).

**Scheme 12:**
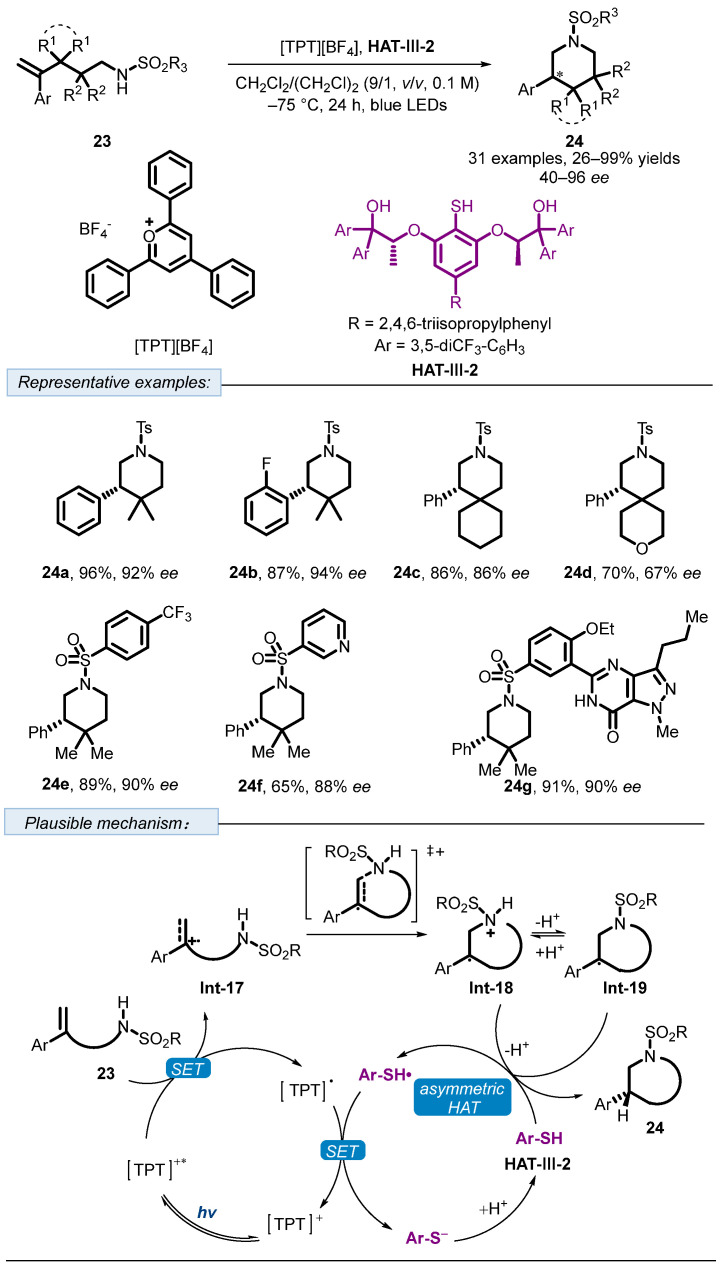
Chiral C_2_-symmetric arylthiol-catalyzed anti-Markovnikov hydroamination. * denotes a chiral center. ‡ denotes a transition state.

In 2024, Dong’s group developed tunable chiral C_2_-symmetric arylthiol catalysts **HAT-III-2** derived from lactate esters, featuring a 2,6-dialkoxythiophenol core with sterically demanding aryl groups (Scheme 12) [41]. These catalysts enabled a highly enantioselective anti-Markovnikov hydroamination-cyclization of *N*-sulfonylated aminoalkenes via visible-light photoredox catalysis, affording 3-substituted piperidines with exceptional enantioselectivity (up to 96%) and yields (up to 99%) at −75 °C. The system exhibited broad functional group tolerance (heteroaromatics, alkenes, alkynes, sensitive CN/OH/OTBS groups) and was applied to late-stage derivatization of drug scaffolds (Celecoxib, Sildenafil, \nx Cholesterol derivatives). Mechanistic studies revealed that the pyrylium photoredox catalyst ([TPT]BF_4_) oxidizes the alkene to a cation radical **Int-17**, which undergoes intramolecular anti-Markovnikov addition to form a prochiral tertiary alkyl radical intermediate **Int-19**. Enantioselectivity is dictated by HAT from the chiral arylthiol, where steric repulsion between the substrate’s *N*-sulfonyl group and the catalyst’s bulky 3,5-(CF_3_)_2_C_6_H_3_ moiety disfavors the (*S*)-enantiomer, leading to the (*R*)-product.

**Scheme 13:**
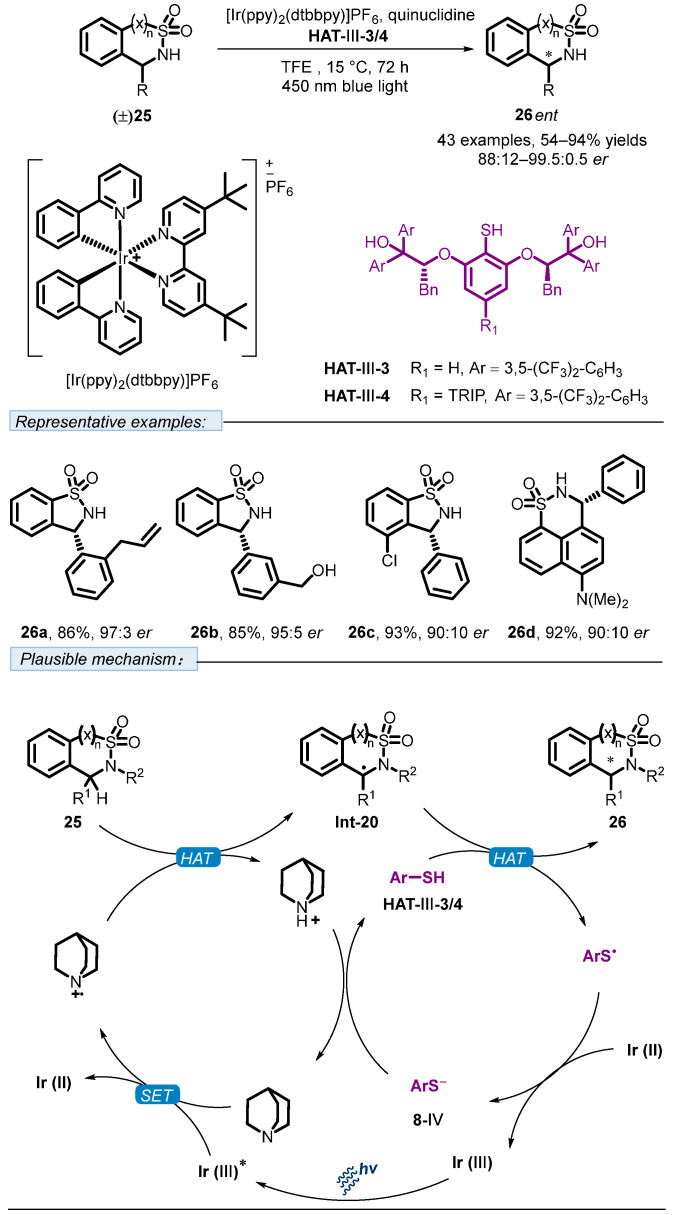
Photocatalytic deracemization of cyclic sulfonamides by quinuclidine and chiral arylthiol catalysis. * denotes a chiral center.

In 2025, Dong’s group and Li’s group established a visible-light-driven deracemization of cyclic sulfonamides using a dual catalytic system comprising an achiral tertiary amine (quinuclidine) and a chiral *C*_2_-symmetric arylthiol **HAT-III-3/4** (Scheme 13) [42]. Under iridium-based photocatalysis ([Ir(ppy)_2_(dibbpy)]PF_6_), the protocol achieves high yields (up to 95%) and enantioselectivity (er up to 99:1) across diverse substrates (aryl-, heteroaryl-, and alkyl-substituted cyclic sulfonamides, sulfamidates, and sulfamates), tolerating sensitive functionalities (aldehydes, alkynes, allyl groups, TBS-protected alcohols). The mechanism involves a sequential relay: photoexcited Ir(III) oxidizes quinuclidine, which abstracts a hydrogen atom non-enantioselectively to generate a prochiral benzylic radical intermediate **Int-20**. The chiral arylthiol then delivers hydrogen enantioselectively via a stereodefining HAT step, governed by sterics and H-bonding (between thiol, ethylene glycol, and sulfonamide). Deuterium-labeling (>99% D-incorporation), Stern-Volmer quenching experiments, and DFT calculations corroborate this pathway and rationalize quinuclidine’s superiority over other amines (e.g., lower barrier for SET). The method was scaled via continuous-flow synthesis (92% yield, 95.5 er) and applied to bioactive motifs (anti-HIV sulfonamides and enantiopure diarylmethylamines). This work expands the toolbox for redox-neutral deracemization via synergistic HAT photocatalysis.

**Scheme 14:**
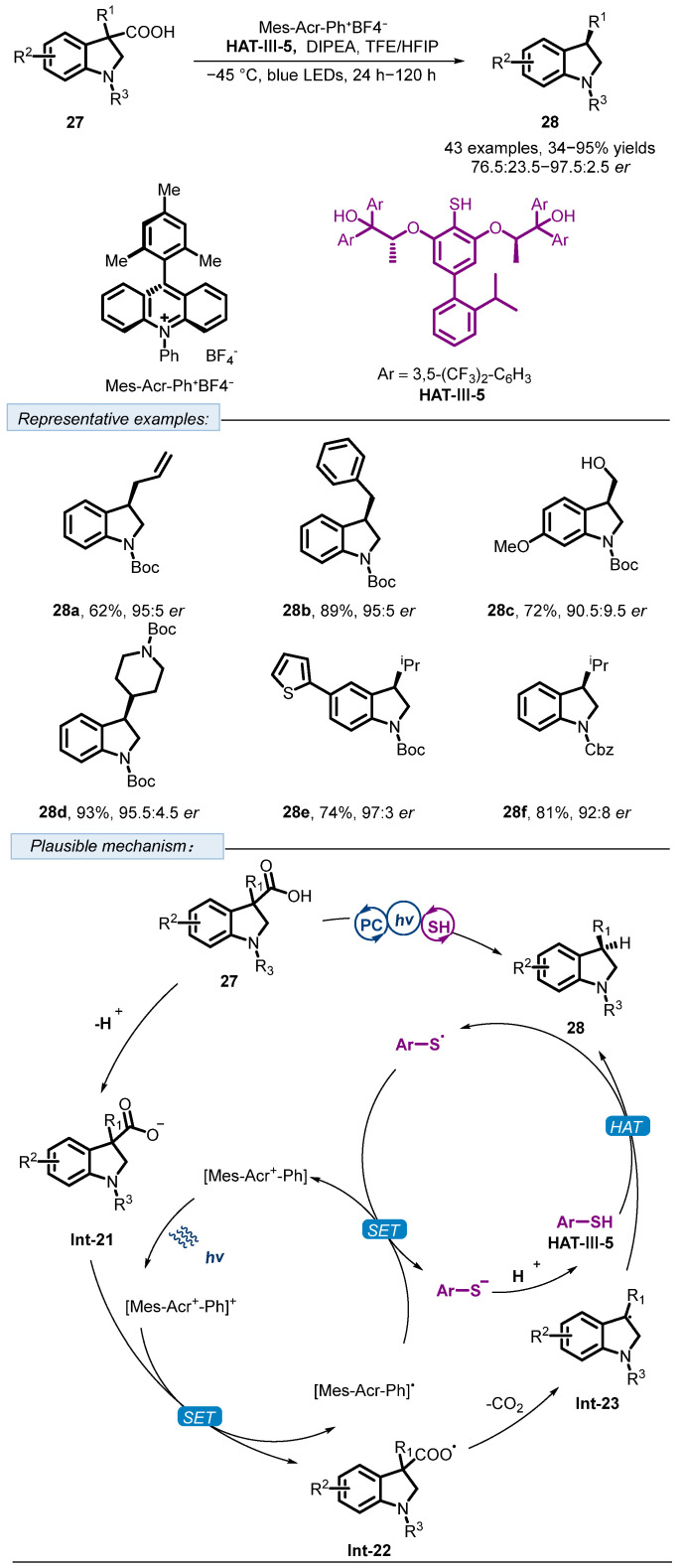
Chiral arylthiol-catalyzed enantioselective decarboxylative HAT process.

In a complementary study, Dong’s group employed a Fukuzumi acridinium photoredox catalyst (Mes-Acr-Ph^+^BF_4_^−^) to oxidize carboxylates, generating a prochiral tertiary carbon radical intermediate (Scheme 14) [43]. The chiral arylthiol catalyst **HAT-III-5** then delivered hydrogen selectively to one face, with enantiodetermination arising from π-π interactions between the catalyst’s 3,5-(CF_3_)_2_C_6_H_3_ group and the substrate’s aryl moiety. Deuterium labeling (using d_1_-TFE) and cyclic voltammetry confirmed the arylthiol as the direct H- source, ruling out chirality memory. DFT calculations identified a favored transition state (ΔG‡ = 3.4 kcal/mol) for *Re*-face attack. This organocatalytic protocol overcomes inherent stereocontrol challenges in radical HAT processes.

## 3. Hydrogen Atom Transfer Reagent of Achiral Thiophenols

In contrast to chiral thiophenols, achiral thiophenols act exclusively as hydrogen donors in HAT reactions, typically affording products without enantioselectivity, under purely kinetic or thermodynamic control. Additionally, examples in which thiol mediates the HAT process by activating R-H substrates, rather than serving as hydrogen donors, are not included.

**Scheme 15:**
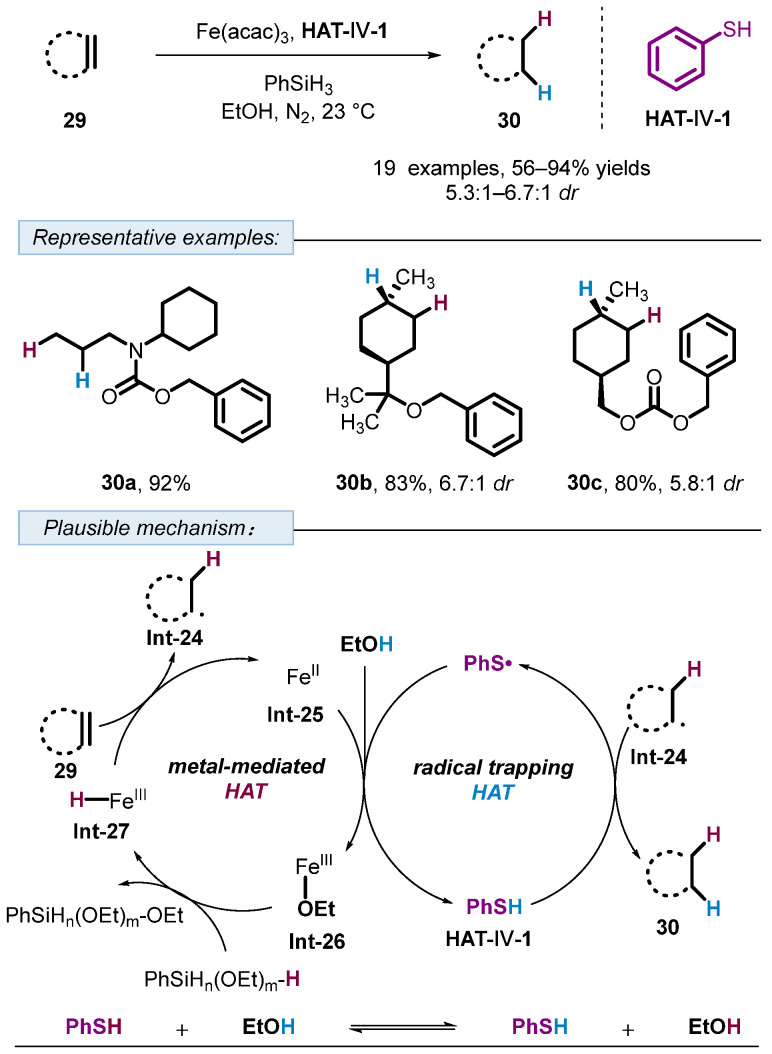
Alkene hydrogenation using non-chiral arylthiol-catalyzed cooperative hydrogen atom transfer (cHAT).

Radical alkene hydrogenation has been extensively studied, and a range of related methods have been reported, making it a valuable strategy for complex molecule synthesis. In 2020, the West group introduced a novel alkene hydrogenation approach based on cooperative hydrogen atom transfer (cHAT) (Scheme 15) [44]. This strategy employs a dual-catalyst system consisting of an iron-based catalyst (Fe(acac)_3_) and a thiophenol **HAT-IV-1** (PhSH), which works synergistically to deliver two hydrogen atoms: one from a hydride (H^−^) supplied by a silane reductant, and the other from a proton (H^+^) provided by a protic solvent. This enables efficient and highly diastereoselective hydrogenation of various unactivated alkenes to the corresponding alkanes without requiring an external stoichiometric oxidant. The method demonstrates excellent substrate compatibility, tolerating functional groups such as amides **30a**, ethers **30b**, and esters **30c**. While the reaction affords thermodynamic products with high diastereoselectivity, no enantioselectivity is induced by the HAT reagent. Mechanistic studies, including isotope labeling, radical trapping, and ring-opening experiments, confirmed the participation of radical intermediates. The process is driven by the coupling of two catalytic cycles: a metal hydride performs the first hydrogen atom transfer to generate an alkyl radical **Int-24**, and a thiyl radical accomplishes the second hydrogen atom transfer to form the product **30**, simultaneously reoxidizing the iron catalyst and regenerating the thiophenol catalyst. Related alkene transformations such as hydrogenation [45], hydrochlorination [46], and azidation [47] have also been reported successively.

**Scheme 16:**
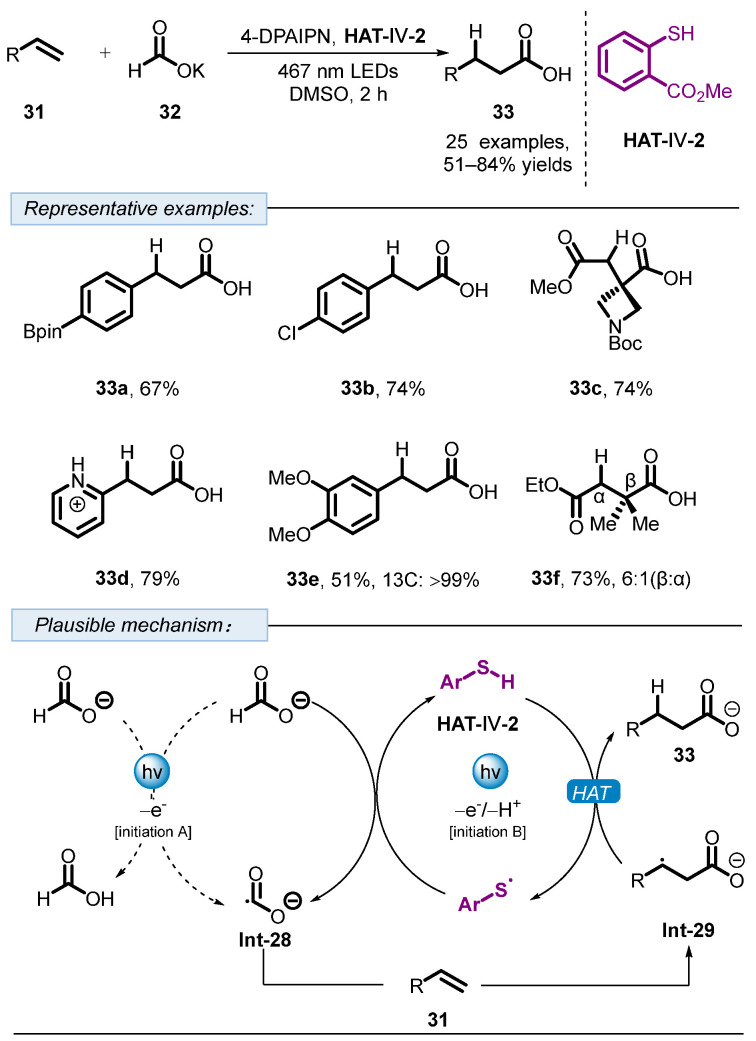
Non-chiral thiol-catalyzed formate delivery for hydrocarboxylation of alkenes.

In recent years, photoredox strategies that generate CO_2_•^−^ in situ via cleavage of the formate C(sp^2^)–H bond have been successively reported [48,49,50], wherein the resulting CO_2_•^−^ serves as a SET reductant. The Wickens group demonstrated the first application of this intermediate in carbon–carbon bond formation. In 2021, they developed a novel photoinduced, thiol-catalyzed, redox-neutral hydrocarboxylation reaction that uses formate as both the carboxyl and hydrogen source, enabling efficient and regioselective synthesis of carboxylic acids from activated alkenes (Scheme 16) [51]. This method features a broad substrate scope, including styrenes with diverse electron-donating, electron-withdrawing, and reductively sensitive functional groups (e.g., boronic esters **33a** and aryl chlorides **33b**), yielding 3-aryl propionic acid derivatives with excellent linear selectivity. It also facilitates the construction of carboxylic acids bearing α-quaternary carbon centers. Furthermore, the reaction offers a practical route to isotopically labeled bioactive carboxylic acids using commercially available labeled formate salts. Mechanistic studies support a thiol-catalyzed radical chain process: the photocatalyst oxidizes thiol **HAT-IV-2** to generate a thiyl radical, which abstracts a hydrogen atom from formate **32** to produce the key CO_2_•^−^ radical anion. This intermediate then adds to alkene **31**, forming a carbon–carbon bond, and the resulting alkyl radical **Int-29** abstracts a hydrogen atom from the thiol catalyst, regenerating the thiyl radical. The reaction proceeds under mild conditions, is operationally simple, tolerates air and moisture, and can be scaled up to the gram scale.

**Scheme 17:**
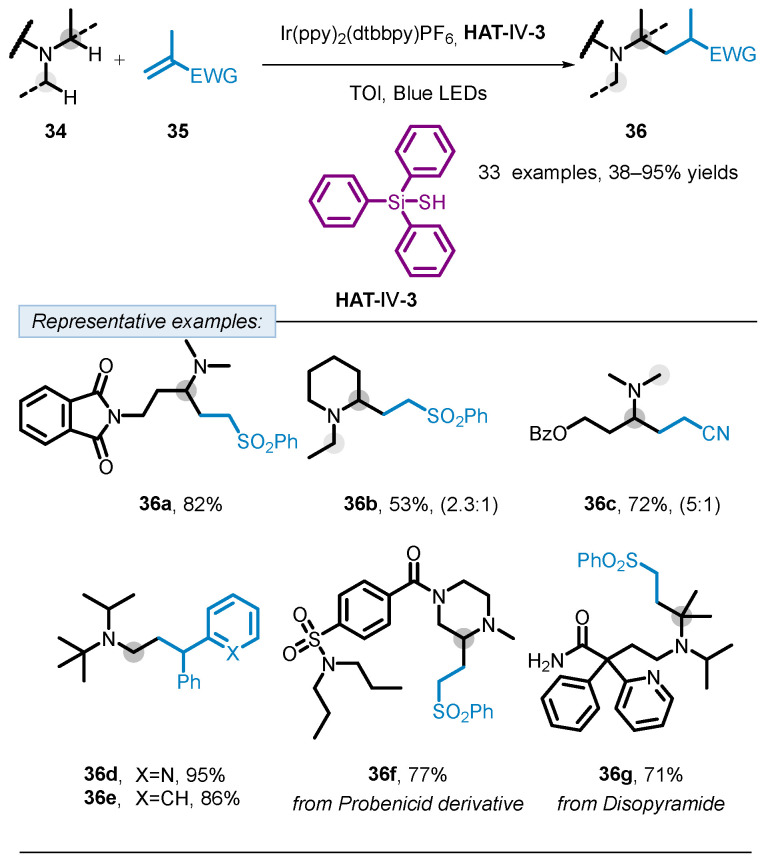
Alkylation of the more substituted a-C(sp^3^)–H bond of trialkylamines.

Traditional approaches for constructing trialkylamines rely on the alkylation of secondary amines or direct C–H functionalization of trialkylamines. However, the α-C–H bonds in unsymmetrical, unfunctionalized trialkylamines often have very similar bond dissociation energies (e.g., N-methylpiperidine: 91 kcal/mol at the secondary site vs. 92 kcal/mol at the primary site) [52,53,54], making selective activation based on inherent enthalpy differences challenging. In 2021, the Rovis and Schoenebeck groups developed a novel strategy based on reversible HAT catalysis that enables highly site-selective functionalization of sterically hindered α-C–H bonds in trialkylamines (Scheme 17) [55]. This approach overcomes the limitation of conventional methods, which typically favor less hindered sites. Using silanethiols **HAT-IV-3** as HAT catalysts under photoredox conditions, a fast and reversible hydrogen atom transfer establishes a dynamic equilibrium of α-amino radicals. Selectivity is governed by the Curtin–Hammett principle, allowing the more substituted and sterically congested α-carbon centers to undergo Giese-type addition with electron-deficient olefins, forming C(sp^3^)–C(sp^3^) bonds. This method shows broad functional group tolerance (including imide **36a**, nitriles **36c**, etc.) and has been applied to the late-stage functionalization of complex drug molecules (e.g., disopyramide **36g**), enabling efficient construction of N-substituted quaternary carbon centers under mild conditions.

**Scheme 18:**
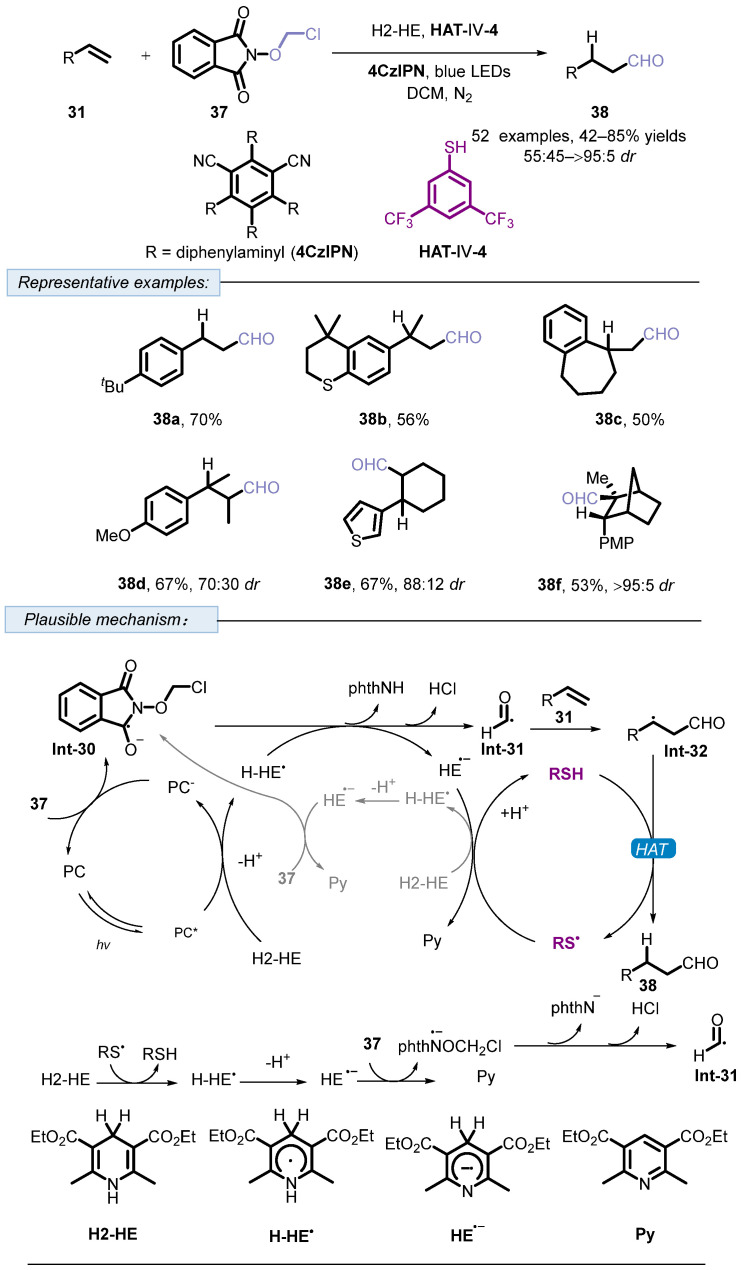
Thiophenol-Catalyzed Radical Hydroformylation of Unactivated Sterically Hindered Alkenes.

In 2025, the Chen and Lan groups reported a thiophenol-catalyzed radical hydroformylation reaction (Scheme 18) [56]. This method achieves efficient and highly regioselective hydroformylation of unactivated, sterically hindered mono-, di-, tri-, and tetrasubstituted alkenes—including electron-rich styrenes and aliphatic alkenes—using readily available and bench-stable α-chloro N-methoxyphthalimide as a formyl radical precursor. A tailored thiophenol-based HAT catalyst (**HAT-IV-4**) significantly lowers the key hydrogen atom transfer barrier (by 8.3 kcal/mol), enabling metal-free synthesis of sterically congested aldehydes that are difficult to access via traditional transition-metal-catalyzed pathways. The mechanism involves photoreductive generation of a formyl radical **Int-31**, which undergoes regioselective addition to the alkene to form an alkyl radical **Int-32**. The thiophenol catalyst then delivers a hydrogen atom, completing the catalytic cycle and regenerating the thiyl radical, while Hantzsch ester acts as the terminal reductant to sustain the radical chain. This radical chain mechanism was corroborated through deuterium labeling experiments, radical clock studies, quantum yield measurements, and DFT calculations.

## 4. Conclusions

This comprehensive review systematically summarizes the emerging applications of thiols/thiophenols and their derivatives as catalysts in HAT reactions. These structurally diverse compounds have garnered considerable attention in organic synthesis due to their unique combination of advantageous characteristics: (1) remarkable structural tunability that enables precise stereocontrol, (2) excellent biocompatibility for potential biological applications, (3) inherent chirality that facilitates efficient enantioinduction, and (4) environmentally benign properties that align with green chemistry principles. Their multifaceted nature positions these sulfur-containing compounds as a privileged class of small-molecule catalysts for radical-mediated transformations. More importantly, the distinctive electronic and steric properties of these organocatalysts offer unprecedented opportunities for developing novel catalytic systems capable of achieving both high efficiency and exceptional enantioselectivity in HAT processes. Future research directions should focus on (i) expanding the substrate scope to include challenging prochiral centers, (ii) elucidating detailed mechanistic pathways to guide catalyst design, and (iii) developing sustainable catalytic protocols. Such advancements will undoubtedly accelerate the construction of structurally diverse chiral molecules with high stereochemical precision, thereby addressing critical synthetic challenges in pharmaceutical development and materials science.

## Data Availability

No new data were created or analyzed in this study. Data sharing is not applicable to this article.
